# Individual curiosity modulates exploration in sequential book selection

**DOI:** 10.1093/pnasnexus/pgag226

**Published:** 2026-06-22

**Authors:** Xuanjun Gong, Erie Boorman, Cuihua Shen, Richard Huskey

**Affiliations:** Department of Communication & Journalism, Texas A&M University, 456 Ross St, College Station, TX 77843, USA; Department of Psychology, University of California, Los Angeles, 1285 Franz Hall, Los Angeles, CA 90095, USA; Department of Communication, University of California, Davis, 469 Kerr Hall, Davis, CA 95616, USA; Department of Communication, University of California, Davis, 469 Kerr Hall, Davis, CA 95616, USA; Center for Mind and Brain, University of California, Davis, 267 Cousteau Pl, Davis, CA 95616, USA

**Keywords:** information seeking, exploration, curiosity, media selection

## Abstract

Information seeking is widely understood as a curiosity-driven exploration behavior similar to resource foraging. However, it remains unclear whether specific exploration decision mechanisms, such as reward generalization and directed exploration, extend beyond physical consumption domains to the semantic spaces of knowledge and information. How do people choose what to read when navigating vast and unfamiliar content landscapes? We study sequential book selection to investigate this question. At times, readers exploit familiar books they expect to enjoy; at other times, they explore novel options to discover potential rewards. Using a large-scale real-world dataset and a controlled behavioral experiment, we show that book selection is guided by structured generalization across a semantic embedding space and by directed exploration toward options with high uncertainty. Moreover, individual differences in curiosity modulate this exploration–exploitation tradeoff, promoting exploratory reading and increasing enjoyment. These findings demonstrate that core computational mechanisms underlying foraging generalize to epistemic domains and shape real-world media selection.

Significance statement How do people choose what to read? Using a large-scale real-world dataset and a controlled behavioral experiment on book selection, we find that people make their reading decisions in ways that resemble food foraging. Readers tend to exploit familiar, rewarding books based on past experiences while also exploring new, unfamiliar options. Crucially, individuals with higher curiosity are more likely to engage in exploratory reading and report greater enjoyment from doing so. This research shows that curiosity shapes how we seek knowledge and entertainment, revealing a fundamental mechanism in human information-seeking behavior.

## Introduction

Information search and acquisition are central to everyday life, shaping how people choose courses, browse websites, read news, and select books. Prior research has conceptualized information-seeking behaviors as a form of foraging (1, 2), that is, searching for high-reward options among many possibilities to achieve optimal outcomes (3, 4). Just as natural foragers seek food resources, people are intrinsically motivated to explore and gather information in vast option spaces like the web or a library (5, 6). To make effective choices, they must address the exploration–exploitation dilemma, that is choosing between options that are known to produce high value or exploring the ones that are novel and unfamiliar for potentially better outcomes. Modern decision theories suggest that people rely on heuristic-based strategies, such as generalization (4), random exploration (7), and directed exploration (8) to solve this issue. However, despite the theoretical link between information seeking and foraging, it remains unclear whether these foraging strategies also govern real-world information-seeking behavior.

Building on the idea that foraging and information seeking, though driven by subjective or epistemic value rather than survival-linked primary rewards, share a common sequential value-optimization structure. Consequently, they may rely on shared decision mechanisms rooted in a common evolutionary origin (2), we examine how people “forage” for books to read. Books, one of the oldest (9) and most widely consumed forms of mass media (10), are used for acquiring knowledge and entertainment (11, 12). Just as foragers seek food by navigating complex environments for optimal reward, readers may similarly navigate vast literary spaces using strategies that balance reward maximization and uncertainty reduction.

Yet, information foraging differs from food foraging in important ways. (i) The option space is effectively infinite (eg the number of published books), (ii) choices are rarely repeated once consumed, (iii) options are embedded in a high-dimensional semantic space rather than a physical one, and (iv) behavior is often driven by intrinsic motivations such as curiosity and interest (13), rather than concrete external rewards like calories or money (14). These differences obfuscate the direct application of exploration–exploitation theories to real-world information-seeking behaviors such as book selection (3, 15).

Recent computational studies (3, 4) have identified that two core algorithmic mechanisms, generalization and directed exploration, can account for foraging behavior in both biological and cognitive domains. Generalization allows individuals to estimate the value of novel options by leveraging structural similarity to past experiences. Directed exploration, in contrast, prioritizes uncertain options that carry high potential for information gain. These mechanisms are particularly relevant to book selection, where readers must choose among many unfamiliar book options with limited reading experiences.

In the context of reading, generalization helps explain how people estimate the value of unread books using prior experience with similar plots or topics. It assumes that options are embedded in a structured feature space, where reward expectations correlate with semantic similarity. Directed exploration explains how readers are drawn to unfamiliar books that evoke curiosity or signal a knowledge gap (14, 16). It posits that people assign an information bonus to options with high uncertainty, inflating their subjective value and motivating exploration.

However, most prior work on these mechanisms has relied on small-sample laboratory tasks or domains involving tangible outcomes like food or money (3). Our study extends this framework to real-world information foraging (ie book selection), where choices are made in large, open-ended environments and rewards are intangible, subjective, and intrinsic. These unique features of information seeking raise important questions about whether generalization and exploration strategies identified in constrained lab settings (4) can also explain how people navigate complex media environments like book selection.

In the current study, we investigate two broad questions. First, we study whether or not different foraging mechanisms, with a focus on generalization and directed exploration, characterize people’s real-world book selection. We approach this question in parallel by investigating people’s learning and selection sequences among two empirical book selection datasets. The first consists of large-scale real-world book selection digital trace data comprised of nearly 35,000 readers and more than 2 million choices (17). These observational data were experimentally confirmed in a second experimental dataset designed to capture the core decision dynamics of book selection. Convergent evidence across two datasets shows that people learn to select more favorable books following a generalization mechanism and are biased toward books with uncertain rewards following a directed exploration strategy. Importantly, by applying these models to book choices—decisions about abstract and information-based resources, we test whether mechanisms developed initially for more concrete, reward-based domains also operate in epistemic decision contexts.

Second, we clarify that the underlying mechanisms of book exploration are modulated by curiosity, referred to as an intrinsic drive for information and learning (18). Prior work shows that curiosity influences preferences for fiction (19), guides attention and information seeking (6, 20–22), and motivates knowledge acquisition and online browsing (5, 23). Therefore, we hypothesized and found that individual differences in curiosity traits regulate people’s book selection. To approach this question, we draw on well-validated conceptualizations that treat curiosity as a personality trait that varies in multiple dimensions (24) to explain variation in people’s exploratory book selection behavior.

The main contributions of this article are 3-fold. First, we introduce a behavioral and computational modeling framework that jointly analyzes real-world digital trace data and controlled experimental data. This approach addresses a complex, domain-specific decision problem—how people choose books—by applying well-established, domain-general theories of sequential decision-making to both naturalistic and high-control settings. In doing so, we also evaluate the extent to which these mechanisms generalize beyond their typical application in constrained laboratory tasks to real-world decisions involving abstract, information-based choices. Second, we show that book selection is regulated by books’ semantic features via a reward generalization mechanism that leads people to select more similar and more favorable books over time. Additionally, people relax semantic feature constraints in their book choices through a directed exploration mechanism in order to deliberately seek unfamiliar books with high uncertainty. Jointly, these selection strategies describe the way people navigate the exploration–exploitation dilemma when choosing what book to read. Finally, we demonstrate that individual differences in random and directed book exploration patterns are explained by the thrill seeking and joyous exploration dimensions of curiosity. In summary, curiosity serves as an intrinsic incentive that boosts book reading enjoyment and encourages reading books with high reward uncertainty.

## Results

We analyzed people’s book selection and rating sequences from a real-world public book review dataset (17) collected on Amazon, one of the world’s largest book purchase and review databases. This dataset consists of 2,083,630 book rating records from 35,478 readers. In addition, we conducted a controlled decision-making task designed to capture the core dynamics of sequential book selection, while eliminating external sources of bias, such as algorithmic recommendations and popularity effects, that are inherent to the Amazon digital trace dataset. This experiment asked participants to make sequential preferential choices among 225 possible book options in a structured grid space. In what follows, we first report the descriptive characteristics of book selections both in real-world and experimental environments embedded in a semantic space. Then, we provide behavioral signatures and computational modeling evidence supporting reward generalization and directed exploration as information foraging mechanisms that govern book selection in both real-world and experimental datasets. Finally, we demonstrate that curiosity modulates these exploration mechanisms in book selection and facilitates enjoyment in book exploration.

### Book selection as exploration in a semantic embedding space

**Figure 1:**
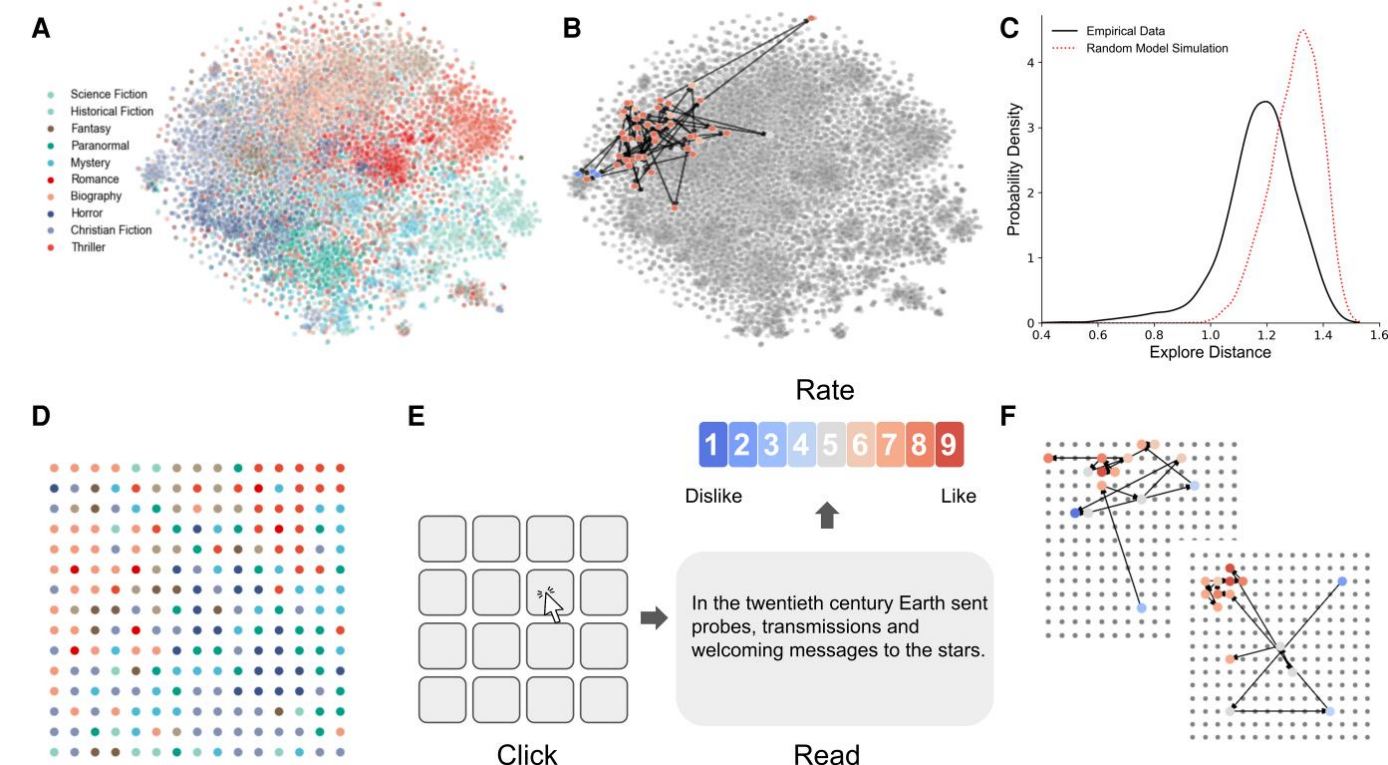
The semantic space of books in the real-world and experimental book selection environment. A) Real-world books are represented in a semantic embedding space. A sample of 10,000 books was plotted in a 2D space, which is t-SNE transformed from a 384D semantic embedding space. Books are naturally clustered by their genre in this semantic embedding space, and together constitute a patchy book foraging environment. B) Readers make sequential book selection trajectories in the semantic book space. Highlighted points depict selected books, with ratings corresponding to the scale shown in Panel E, while faint background points represent books available but not selected. The arrows connecting two points denote the sequential order of consecutive book choices. C) Probability density plot of the distribution of people’s exploration distance (solid line curve), which is measured as the Euclidean distance between the semantic embedding vectors of consecutive book choices. The dotted line curve denotes the null distribution of exploration distance, which assumes people randomly select books. D) The experimental book selection landscape. A total of 15 × 15 options were arranged in a grid and presented for participants to make book selections. Each point encodes a book option, and each point is visually encoded to indicate the genre of the book. Book options were arranged so that semantically similar books are placed close to each other. E) Participants completed a total of 15 trials of a click-read-rate task, where they clicked one option from the grid, read the synopsis of the book, and then rated their reading enjoyment. F) Participants’ sequential book selection trajectories in the experiment.

In real-world book selection, the number of book options is enormous, and these books differ from each other in many ways. Conceptually, books are embedded in a semantic space such that each book can be represented by its semantic features. To reconstruct this semantic space for books included in our dataset, we collected the synopsis for each book option from the GoodReads website, one of the largest book metadata databases. We then transformed the summary for each book into a multidimensional semantic embedding vector using an advanced natural language processing technique (see Materials and methods, Fig. 1A). This embedding represented all books in the real-world Amazon dataset, and a curated subset of 225 books for the experimental dataset (see SI Appendix for details). To test the validity of this semantic embedding method, we collected perceived pairwise dissimilarity ratings among a subsample of the 225 book options selected for the experimental dataset (n=22 unique options; 10%) from 248 participants recruited from Prolific. A Mantel test (25) shows a significant positive association between human-perceived dissimilarities and machine-evaluated semantic dissimilarities (r=0.536,Z=7.248,P<0.001; all significance tests were two-tailed; see SI Appendix for full reports).

Previous works widely consider that people’s exploration decisions resemble foraging behavior in a patchy environment (4, 26), which exhibits a clumpy spatial distribution of resources (27). Consistent with this hypothetical idea, we found that book options are naturally clustered in a patchy format and grouped by their genre in semantic space (Fig. 1A). To measure to what extent books are clustered in the semantic embedding space, we calculated the Hopkins’ Statistic as a measure of the clustering tendency of all books (28) against a null uniform spatial distribution (a Poisson point process). Using a random sample of 5% of the dataset, we found book options have a clustering tendency (H=0.754) exceeding standard heuristic thresholds at 0.75 (29), which indicates that book options are clustered rather than randomly dispersed in the embedding space.

In this embedding space, book selections can be ordered as a sequence of nonrepetitive discrete choices represented by a set of numeric semantic features (Fig. 1B). We found that people’s book explorations were constrained by semantic distance among options. The observed distances between consecutive choices (M=1.170,SD=0.155) are significantly smaller (Z=−1,172.34,P<0.001; Fig. 1C) than the baseline semantic distances (M=1.301,SD=0.090), which were calculated using all pairwise distances among a random sample of 10,000 book options drawn from the dataset. Thus, readers are more likely to explore a book that is semantically similar to previously read books compared to randomly chosen alternatives, which is consistent with previous research on memory retrieval and purchase behaviors (3, 30).

These results depict people’s book selections as a trajectory of explorations in a patchy environment embedded in a multidimensional semantic space, where people decide which book to read based on the semantic features of previously read books and available book options. We formalized this decision problem using a multiarmed bandit task, a widely used experimental paradigm for studying exploration decisions (31), to capture the essential decision dynamics of book selection within a controlled environment (4). In this task, a 15 × 15 2D grid was displayed, with each cell representing one of 225 unique book options selected from the real-world dataset (Fig. 1D). The pairwise spatial distances among options in this grid space were designed to represent the semantic distance among the corresponding books in a way such that semantically similar options were placed close to each other and grouped in patches. Using this experimental paradigm, we collected sequences of book choices and reading enjoyment ratings from 250 participants (Fig. 1E) and conducted further analysis to explore people’s sequential book selection patterns (Fig. 1F).

### Reward generalization guides book selection

**Figure 2:**
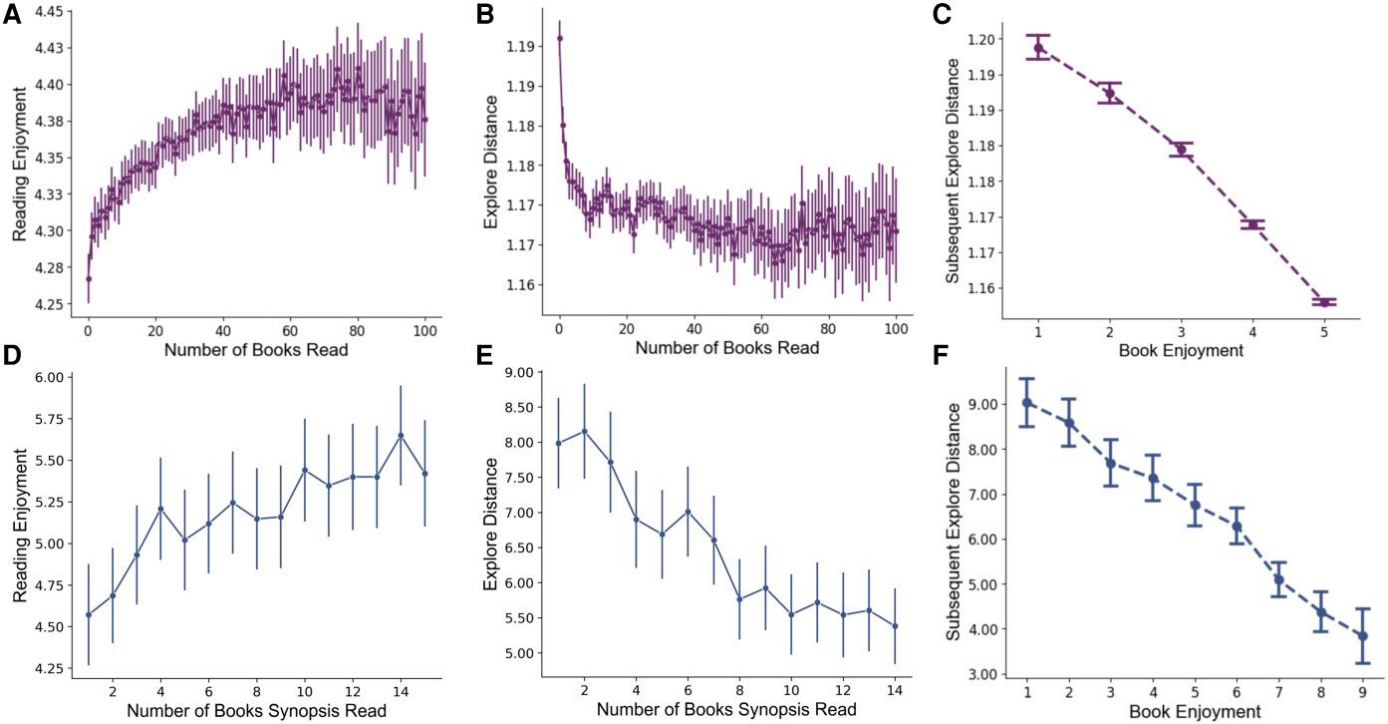
Signatures of learning and reward generalization in real-world and experimental data. Top row A–C): Real-world Amazon dataset. Bottom row D–F): Controlled experiment dataset. A, D) Average reading enjoyment increases as a function of the number of books read. B, E) The exploration distance, measured as the Euclidean distance between semantic embedding vectors of consecutive book choices, by the number of books that have been read. C, F) The relationship between immediate reward and subsequent exploration: higher ratings for the preceding book are associated with smaller exploration distances in the subsequent choice. The points in the line plot indicate the mean estimates, and the vertical lines indicate the 99% CI. Note: distances are in arbitrary units, as Euclidean distances calculated within their respective spaces: a 384D semantic embedding space for the Amazon dataset and a 15 × 15 spatial grid for the experimental dataset.

We assessed whether or not people learn to make better book choices over time in both datasets. Book ratings in the real-world datasets range from 1 star (lowest rating) to 5 stars (highest rating), while ratings in the experiment were measured by a 9-point scale ranging from 1 (extremely disliked) to 9 (extremely liked). To investigate the bivariate relationships between variables, we fit simple linear regression models with mixed effects, including random intercepts and random slopes for individual readers to control for individual variability, and report the standardized regression coefficients *β* as estimates of the strength of the bivariate relationship. We found an increasing linear trend in people’s reading enjoyment over time (Fig. 2A and D) in both real-world (β=0.052,  SE=0.002,Z=25.357,P<0.001,95  0.056]) and experimental (β=0.107,SE=0.017,Z=6.274,P<0.001,95  CI=[0.073,0.140]) datasets. The increased enjoyment indicates that people learn to choose more favorable books over time. (See SI Appendix for alternative explanation tests (15).)

In addition, we looked at the evolving patterns of people’s book explorations as a function of semantic distance between consecutive choices. We found that people’s book exploration stabilized over time for both the real-world (β=−0.009,  SE=0.002,Z=−4.872,P<0.001,95) and experiment datasets (β=−0.195,SE=0.023,  Z=−8.419,P<0.001,95). This indicates that, over time, readers tend to select books that are increasingly semantically similar to their previous choices (Fig. 2B and E). Combined, people make more favorable and similar choices over time, indicating that they learn from previous book-reading experiences, gain better value estimations of book options, and choose high-value books accordingly. This mechanism is consistent with reward generalization (32), by which people use feature similarities to update their reward estimations as of function learning (31).

Importantly, reward generalization formalizes that, after a high-reward book reading experience, people estimate similar books to have higher values and do the contrary after a low-reward experience. Therefore, people tend to choose books that are similar to previously experienced high-reward books while avoiding books similar to low-reward books. Consistent with this prediction, we found that people tend to choose semantically similar books after a high-reward relative to a low-reward reading experience (real world: β=−0.040,SE=0.001,Z=  −47.310,P<0.001,95; experimental: β=−0.335,SE=0.019,Z=−17.584,P<0.001,95  −0.298]; Fig. 2C and F). Thus, we found evidence indicating that readers employ a reward generalization strategy in book selection behaviors, which helps them quickly learn which option generates high rewards, discover their favorite book types, and improve their overall reading experiences.

### Directed and random exploration

People do not always choose books from their favorite genres. Sometimes, people select unfamiliar books and explore novel genres. This behavioral pattern suggests another critical exploration mechanism that might govern book selection—directed exploration, which involves deliberately seeking books with high novelty and uncertainty to gain knowledge while forgoing the immediate rewards of reading familiar books. Contrary to random exploration strategies, which specify that people’s exploration is passively driven by the stochasticity of the decision-making process, directed exploration hypothesizes that people actively add an information bonus to the reward estimation of high-uncertainty options in order to encourage choices toward uncertain options.

**Figure 3:**
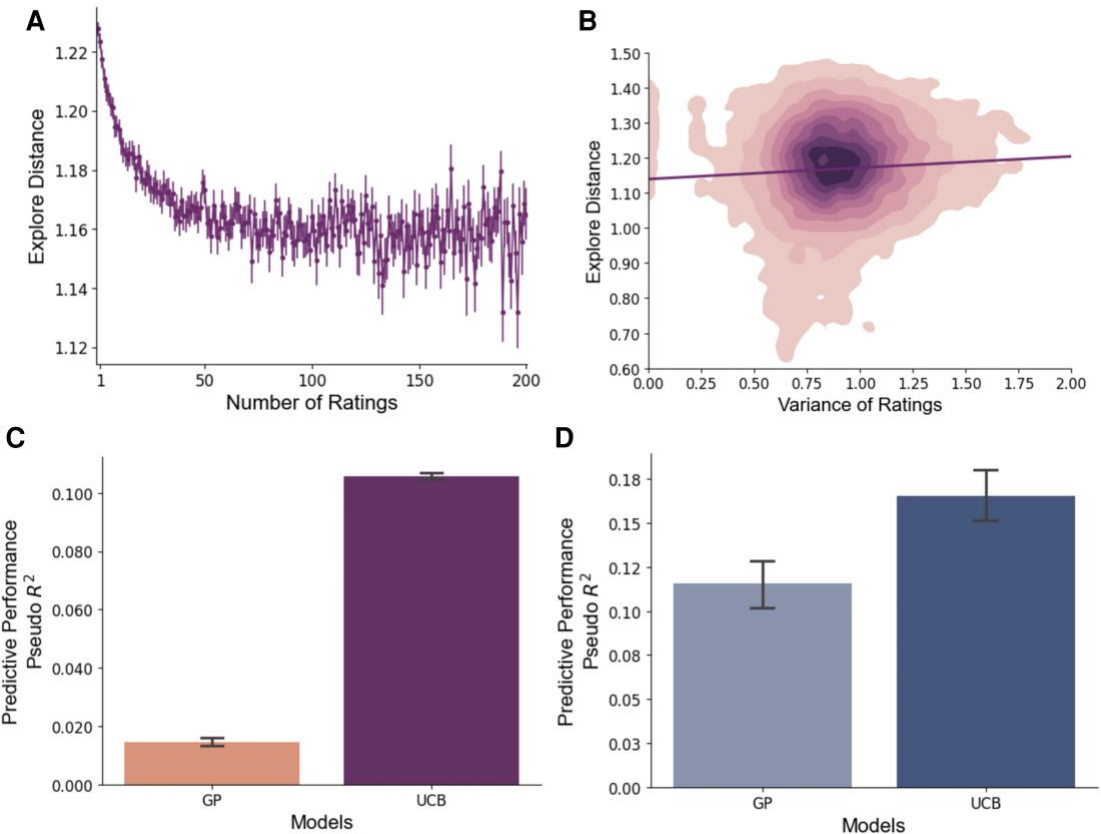
Signatures of directed exploration in the real-world data, and model prediction accuracy in both real-world and experiment data. A) Exploration distance by the number of ratings for subsequent book choices. Mean estimates were plotted as points, and 99% CI were plotted as vertical lines. B) Bivariate distributions of exploration distance and the variance of ratings for subsequent book choices. Darker colors indicate higher probability density. The solid line is plotted as the regression line of variance of ratings regressing on exploration distance. The predictive accuracies for the GP and the UCB models for each subject are plotted for real-world dataset (C) and experimental data (D). Bar height indicates a mean estimate for the predictive accuracy (measured as pseudo-$R^{2}$) for all subjects in the dataset, and the vertical line indicates the 99% CI of the predictive accuracy.

We operationalized book uncertainty by evaluating the number of ratings and the variance of ratings (33) in the GoodReads metadata. Although Goodreads metrics were not directly visible on Amazon product pages at the time readers made their purchase decisions, they were easily accessible to the readers and served as robust proxies for the general enjoyability and public familiarity of the selected books. Compared to books that have rarely been rated or have heterogeneous ratings, books with a large number of homogeneous ratings should give readers more confidence in estimating their reading rewards and, hence, have lower uncertainty. Indeed, people’s book exploration distance is associated with the total number of ratings and rating variance of the selected book (Fig. 3A and B). We found a significant negative relationship between the logarithm of rating counts and exploration distance (β=−0.053,SE=0.001,  Z=−52.610,P<0.001,95), as well as a significant positive correlation between rating variance and the exploration distance (β=0.026,SE=0.002,  Z=12.550,P<0.001,95). We noticed that books’ average ratings are negatively correlated with their rating variances (r=−0.485,P<0.001). A sensitivity analysis confirmed that the relationship between rating variance and explore distance remains robust even when controlling for the books’ average ratings (see SI Appendix). Furthermore, the mixed-effect model that includes all four independent variables (ie number of past reads, preceding reading rating, logarithm of rating counts, and rating variance), random intercept and random slopes revealed consistent results with our bivariate statistical results across both datasets (see SI Appendix). Additional sensitivity analysis focusing on different data filtering criteria and distance metrics yield consistent results (see Materials and methods and SI Appendix).

### Computationally modeling sequential book selection

We further evaluated one-step-ahead prediction accuracy (ie the ability to predict the next choice based on previous choices across the decision trajectory) of the corresponding computational models of the generalization and directed exploration mechanisms for individual book selection sequences (Fig. 3C and D).

First, to capture generalization, we employed a Gaussian Process (GP) regression model. Simply put, the GP model predicts how much a user will value a new book by calculating its semantic similarity to books they have previously enjoyed. Conceptually, the GP model assumes that learners generalize their value expectations over the semantic space: if a user enjoys a specific book, the model predicts they will expect high value from semantically similar books (4, 34). Thus, the GP model represents a pure exploitation combined with a random exploration strategy, where choices are driven solely by expected value with random noise. This model relies on two key parameters: a generalization parameter (*λ*), which determines how broadly a reader applies past experiences to new, semantically distant books, and a temperature parameter (*τ*), which captures random exploration such that higher *τ* leads to a stronger tendency toward random exploration.

Second, to capture directed exploration, we utilized an Upper Confidence Bound (UCB) model. The UCB model builds upon the GP but adds an explicit uncertainty bonus to the value estimation. It introduces a directed exploration parameter ($\beta_{\mathrm{bonus}}$) that quantifies how much a reader is intrinsically drawn to uncertainty. It predicts that users are driven not just by high expected reward, but also by high semantic uncertainty, by actively seeking out books that are semantically distinct from their previous reading history to gain new information (3, 4).

Accuracy was computed as model performance normalized against a null random model (4). For the experimental dataset, we compared the likelihood of the selected book to all available alternatives. For the real-world dataset, where the choice set is unbounded, we followed (3) and compared the likelihood of the observed choice to a null option with averaged semantic features (see SI Appendix for full model specification).

The GP model, incorporating generalization and random exploration, outperformed chance in both datasets (real-world: $R^{2}=0.015,Z=30.011,P<0.001,99\;$; experimental: $R^{2}=0.116,Z=17.024,P<0.001,99\;$  0.133]). Adding a directed exploration term (information bonus) further improved prediction in the UCB model (real-world: $R^{2}=0.107,Z=512.134,P<0.001,99\;$; experimental: $R^{2}=0.165,Z=12.984,P<0.001,99\;$  0.185]).

Together, these results support the two key foraging mechanisms: (i) generalization, where readers learn from prior experience to evaluate books by semantic similarity and (ii) directed exploration, where uncertainty increases a book’s subjective value, encouraging information-seeking in book selection behaviors.

### Curiosity modulates exploration in book selections

Epistemic curiosity is a core intrinsic motivator for uncertainty reduction and knowledge acquisition (6, 14, 35, 36). Empirically, curiosity promotes information seeking through media use (37) and helps explain individual differences in media consumption patterns (1, 5, 24). Therefore, we asked whether trait curiosity characterizes individual variability in book exploration.

**Figure 4:**
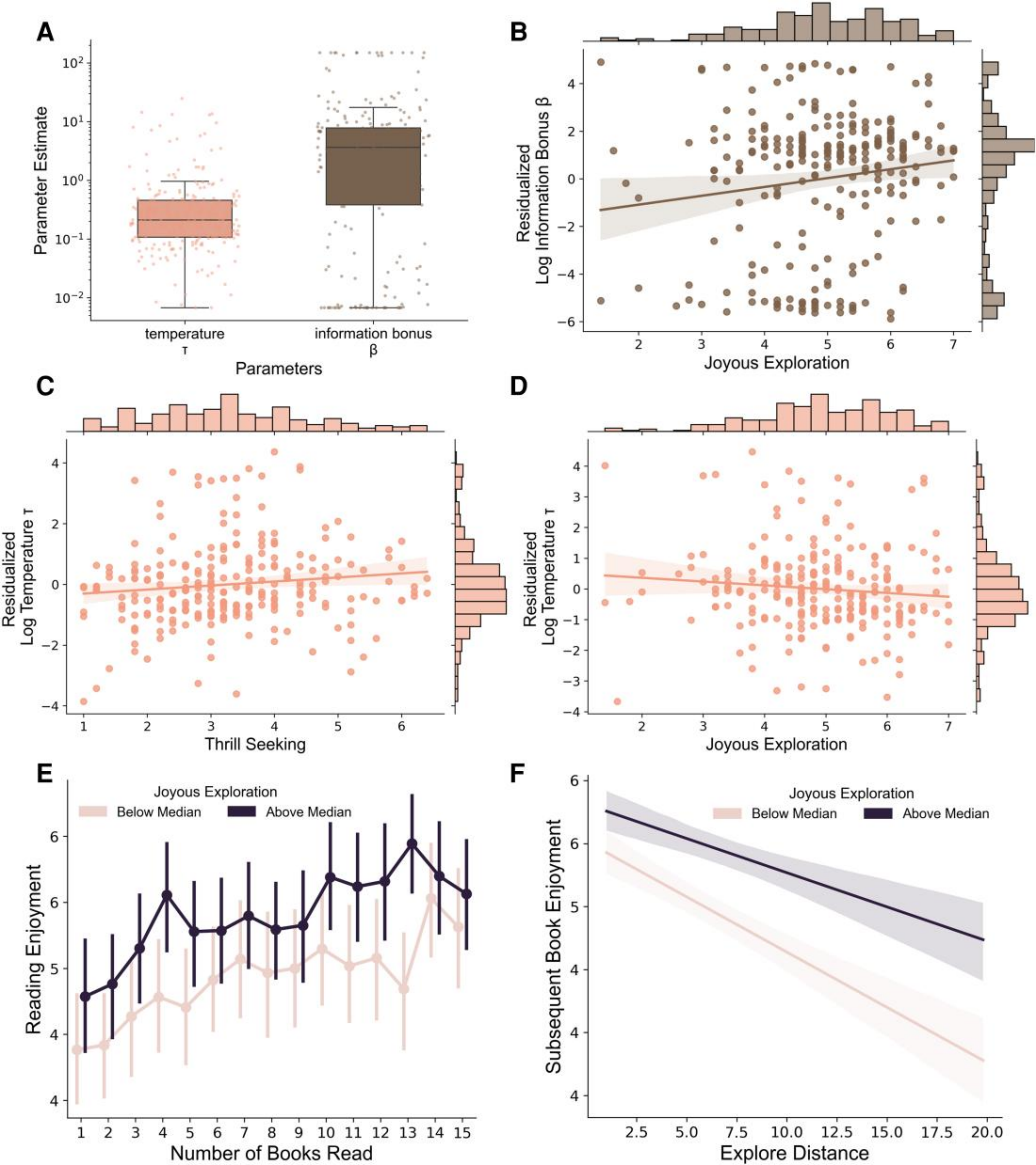
Curiosity modulates exploration patterns in people’s book selection. A) Boxplots of cross-validated parameter estimates of the temperature parameter *τ* (left box plot) and the information bonus parameter $\beta_{\mathrm{bonus}}$ (right box plot) for each participant in the experimental dataset. Parameter estimates for each participant are shown as points displayed for each box plot and are horizontally jittered for improved visual interpretation. B) The partial regression plot of the logarithm of $\beta_{\mathrm{bonus}}$ regressed on joyous exploration; C) logarithm of *τ* regressed on thrill seeking; and D) logarithm of *τ* regressed on joyous exploration. The solid line indicates the estimated regression line, and the shaded area around the regression line represents the 95% CI of regression coefficient estimates. E) Line plot of reading enjoyment by the number of books that have been previously read in the experimental dataset, for participants high (darker colored line) and low (lighter colored line) in joyous exploration. Mean estimates are plotted as points, and 95% CI are plotted as the vertical lines. F) The regression models of subsequent book enjoyment regressed on exploration distance for participants high (darker colored line) and low (lighter colored line) in joyous exploration.

To answer this question, we identified computational phenotypes (38) of exploration decisions by estimating the temperature parameter (*τ*), indexing random exploration, and the information bonus parameter ($\beta_{\mathrm{bonus}}$), indexing directed exploration, for each participant independently (Fig. 4A). We identified a moderate positive correlation between *τ* and $\beta_{\mathrm{bonus}}$ (r=0.193,P=0.002). We then regressed the log-transformed values of *τ* and $\beta_{\mathrm{bonus}}$ on a 5D trait curiosity scale-measuring joyous exploration, thrill-seeking, stress tolerance, deprivation sensitivity, and social curiosity (24), while controlling for age, a known predictor of exploratory behavior and media use patterns (39, 40).

Directed exploration ($\beta_{\mathrm{bonus}}$) was positively associated by joyous exploration (Fig. 4B; β=0.229, SE=0.081, t(238)=2.824, P=0.005, 95). In contrast, random exploration (*τ*) was positively associated with thrill-seeking (Fig. 4C; β=0.200, SE=0.082, t(238)=2.440, P=0.015, 95) and negatively associated with joyous exploration (Fig. 4D; β=−0.160, SE=0.080, t(238)=−1.983, P=0.049, 95; see SI Appendix for details). Together, participants high in joyous exploration (the desire to seek out and take joy in new knowledge) practice less random but more directed exploration, suggesting a strong preference for books with high uncertainty by systematically assigning an information bonus to the uncertain books when evaluating their subjective value. On the other hand, participants high in thrill-seeking (the willingness to take risks in order to achieve high-variance experiences) have a stronger tendency to adopt a random exploration book selection strategy instead of following the directed exploration strategy. Importantly, these systematic correlations with stable personality traits, combined with normally distributed parameter estimates lacking extreme ceiling or floor effects (see Fig. 4C and D), suggest that *τ* captures a genuine, computationally meaningful exploratory behavior rather than merely inattentive or noisy task responding.

Joyous exploration modulates people’s directed exploration behaviors by attaching intrinsic incentives to high-uncertainty options. Following this idea, we looked at people’s reward experiences in book exploration as related to their joyous exploration. We found that people with high joyous exploration (above a median split) generally experienced higher reading enjoyment compared to people with low joyous exploration (below a median split; Fig. 4E). Furthermore, we identified that this difference in reading enjoyment mainly arises from reading semantically different books rather than semantically similar books (Fig. 4F). A mixed-effect regression model with reading enjoyment as a dependent variable revealed a positive main effect of trait joyous exploration (β=0.146,SE=0.034,Z=4.347,P<0.001,95  CI=[0.080,0.212]), and a positive interaction effect between the semantic similarities between consecutive books and people’s joyous exploration trait (β=0.033,SE=0.017,  Z=1.990,P=0.047,95; see SI Appendix for full results). Thus, compared to people with low joyous exploration, people with high joyous exploration gain higher rewards from their exploratory book choices. This result echoes previous studies showing that curiosity positively boosts experienced enjoyment during information-seeking (23, 41).

We conducted linear regressions on directed and random exploration after removing nonsignificant covariates, and the results remain robust (see SI Appendix). We note that a considerable portion of the directed exploration parameter estimates are at a boundary close to zero. We consider this a feature rather than a bug because this near-zero parameter estimate captures meaningful exploration characteristics for nonexploratory participants. To verify the robustness of the regression results, we fit a censored regression model specifying a left boundary near zero, and the results remain consistent (see SI Appendix). Additional robustness checks were carried out to evaluate the stability of the parameter estimation, given that the global optimization method is nondeterministic. We repeated the optimization method 100 times independently, and parameter estimates for each decision parameter and each participant were reliably stable (see SI Appendix).

## Discussion

People forage for information resources such as books. This similarity arises as both domains represent instances of the same general search task (2, 42). While the nature of the reward differs, the computational structure remains identical: an agent must identify optimally valued options among numerous alternatives through learning and exploration.

Building on this theoretical framework, we investigated how individuals make sequential information-foraging decisions using both a large-scale real-world book selection dataset and a controlled behavioral experiment. We found that information-seeking and resource foraging rely on similar decision mechanisms, likely stemming from a common evolutionary foundation. First, book options are distributed in a patchy semantic space, and readers’ choices are constrained by the semantic distance between consecutive selections. Second, book choices align with computational reinforcement learning mechanisms previously applied to food foraging (3), specifically a generalization mechanism, by which people learn from past reading experiences to guide future selections, and a directed exploration mechanism, which promotes sampling of unfamiliar, high-uncertainty options. Finally, we found that trait curiosity, particularly the joyous exploration and thrill-seeking dimensions, modulates book selection by encouraging directed exploration and enhancing enjoyment, especially for semantically novel books.

These results provide empirical support for the generalizability of domain-general foraging mechanisms to information-seeking behaviors. Book choices are epistemic in nature, oriented toward acquiring knowledge, insight, or entertaining experience rather than satisfying material needs. These decisions are typically nonrepetitive, made within vast option spaces, and occur within a structured semantic environment. By incorporating trait-level curiosity and validating computational patterns across both naturalistic and experimental contexts, our study extends the scope of these decision-making mechanisms to intrinsically motivated, semantic domains. This work clarifies how exploration strategies adapt in environments where outcomes are defined by their epistemic value. An important avenue for future research will be determining the boundary conditions of these foraging principles. We hypothesize that these mechanisms should robustly generalize to other goal-directed, value-optimization domains like movie selection or deliberate news consumption. Conversely, they may operate differently in highly unstructured environments driven by pure, unbounded curiosity, such as traversing Wikipedia hyperlinks (5) or scrolling through infinite social media feeds.

Admittedly, real-world information-seeking choices are inherently complex and likely involve cognitive mechanisms beyond those investigated in this study, as reflected by the smaller effect sizes observed in the real-world dataset compared to the experimental one. Applying simple cognitive models to real-world decision-making presents substantial challenges, reflecting a systematic discrepancy between complex real-world decision contexts and an idealized theoretical decision environment. This discrepancy is largely attributable to the vast dimensionality of the real-world decision space, where users select from a large number of available items, compared to the constrained option sets typical of laboratory tasks, as well as substantial contextual differences between economic decisions and information foraging decisions. In contrast, modern media recommendation systems often rely on large-scale, black-box models—such as deep neural networks—to achieve high predictive accuracy (43). While effective, these models are typically uninterpretable and offer limited insight into the cognitive processes that drive information-seeking behavior. Interpretable cognitive mechanisms, like those validated here, have already begun to inform the design of more transparent systems. For instance, curiosity-informed recommendation algorithms (44) may offer promising avenues for future development.

We acknowledge that both our real-world digital trace data and experimental behavioral data are subject to specific limitations. Real-world digital traces are often skewed toward highly active users and contain environmental confounders inherent to the platform interface, such as algorithmic recommendations, popularity sorting, and the visibility of other readers’ reviews. These external factors make it difficult to analytically disentangle intrinsic user preferences from platform-driven nudges. On the other hand, behavioral data from the controlled experiment allowed us to isolate decision mechanisms in a neutral environment free from algorithmic recommendations or social cues, though it may not perfectly replicate real-world decision scenarios due to the artificial task environment. However, utilizing both real-world and controlled data allows us to triangulate our findings and mitigate their own limitations. The convergence of directed exploration patterns in both datasets suggests that the behaviors observed in the Amazon platform are likely driven, at least in part, by organic user agency rather than platform artifacts, thereby supporting the internal and ecological validity of our conclusions.

Finally, our study relies on the correlation between trait curiosity and computational exploration parameters. While this approach follows established frameworks in computational phenotyping and decision science (5, 38, 39) to demonstrate phenotypic validity, it does not strictly isolate causal mechanisms. It remains possible that an unmeasured third variable influences both self-reported curiosity and semantic exploration. Future work should aim to test the causal link by experimentally manipulating curiosity states (eg via curiosity-eliciting strategies (45)) to observe consequent changes in exploration parameters.

Extending computational decision models to a broader range of real-world environments offers a promising direction for future research. While our study focused specifically on book selection as a form of information seeking, we anticipate that similar patterns may extend to other domains, such as social media engagement, movie selection, website browsing, and news consumption. Prior work has already shown that social media use can be modeled using reinforcement learning mechanisms (46), and that Wikipedia browsing behavior is shaped by semantic link structure and modulated by individual differences in trait curiosity (5). However, the extent to which generalization and curiosity-driven directed exploration apply across diverse forms of real-world information seeking behaviors remains an open empirical question. Future empirical studies could investigate how these mechanisms operate across different media environments. In parallel, theoretical efforts would benefit from considering the constraints and affordances of real-world decision contexts. Doing so will not only test the boundaries of current decision models but also enhance their practical relevance and guide the development of next-generation theories of decision making.

## Materials and methods

### Ethical statements

The Institutional Review Board at the University of California, Davis provided ethical approval for the study protocols of the embedding validation study and the behavioral experiment. The methods were carried out in accordance with the relevant guidelines and regulations. All participants provided informed consent prior to participating in the study.

### The Amazon dataset

This dataset consists of a representative subset of engaged readers’ book selections and ratings on Amazon (17). It contains 35,478 readers, leaving 2,083,630 reading and rating records for 416,797 books. Each reader left 59 (SD = 40) book reading and rating records on average. We arrived at this dataset by filtering out readers who left fewer than 30 records or more than 300 records. Doing so helps us maintain a reasonable horizon length that is long enough to probe learning and exploration dynamics, while not too long to demand unaffordable computational expense (sensitivity analysis on users with fewer rating records yields consistent results, see SI Appendix). Additionally, we filtered out readers for whom more than 10% of records were placed at the same timestamp, a step necessary to address the “timestamp collision” problem (47) and remove potential batch review activity. An additional sensitivity analysis focusing on verified rating records yields results consistent with our main findings (see SI Appendix). We collected the book metadata, including synopsis, rating distributions, and genres from GoodReads, and reading records without corresponding metadata were excluded (N=28,303;1.3 of all records).

### Book semantic embedding

We created a latent semantic space for book embeddings. First, we preprocessed the book synopsis texts by removing HTML tags, nonalphanumeric characters (eg punctuation and symbols), and excessive white spaces. Then, we encoded the preprocessed book synopsis into 384D embedding vectors for each book, using the state-of-the-art sentence-transformer model All-minilm-l6-v2 (https://huggingface.co/sentence-transformers/all-MiniLM-L6-v2). Thus, the pairwise semantic distance between books was calculated as the Euclidean distance between their semantic embedding vectors (a sensitivity analysis using cosine distance metrics yields consistent results; see SI Appendix). Distinct from previous studies (3, 15), which measure the frequency of nonrepetitive choices as indices for exploration, book choices are nonrepetitive in nature. Thus, we measured the extent of exploration, captured by the semantic distance between consecutive choices, as an valid way to quantify people’s book exploratory selections.

### Embedding validation

To verify the validity of the semantic distance measures, we asked 248 participants (131 female; M ± SD age: 40±13 years), on Prolific to rate the perceived pairwise similarities among 22 randomly sampled books. These 22 books give a total of 231 combinations of book pairs to be evaluated. Each participant was paid $4.47 (equivalent to $12/hour) to evaluate similarities of 15 randomly sampled book pairs after reading the book synopses for both books. Similarity was evaluated on a scale ranging from 1 (extremely dissimilar) to 9 (extremely similar). On average, each pair of books received 16 ratings. Given this randomized design, we assessed the reliability of the mean ratings ($\mathrm{ICC}(1,k_{\mathrm{rater}})$ (48)) using a linear mixed model; the resulting coefficient of 0.87 indicated strong stability of the consensus scores. Finally, we took the averaged similarity ratings for each book pair to test the validity of the semantic distance measures from the embedding method. We constructed two distance matrices, one with the Euclidean distance metrics in semantic space and the other with participant evaluated similarities. Then, a comparison between these two distance matrices was conducted using the Mantel test (25), which evaluates the association between distance matrices while accounting for the inflated number of observations of pairwise distances.

### The experimental dataset

Participants (n=250) were recruited from Prolific and paid $6 (equivalent to $12/h) for their time (31.6±15.9 min) in the experiment. Participants (*n* = 5) who failed the attention check were excluded from the analysis, thus resulting in a final sample size of 245 (129 female; M±SD age: 40±13 years).

### Stimulus preparation

Book selection resembled a multiarmed bandit task that simulated the real-world book selection environment. We first selected a subset of 225 books (see SI Appendix for details), which includes the 22 books used for semantic embedding validation, from the real-world dataset. Then, we applied a multidimensional scaling technique (49) to project each book synopsis’s 384D embedding vectors down into 2D vectors. This dimensionality reduction method maximally preserves the pairwise distances between books from high- to low-dimensional space. Next, we arranged these book options into a 15 × 15 grid based on their 2D embedding vector in a way such that the Euclidean distance on the grid represents the semantic distance between books. This grid setting was identical for all participants in the experimental setting.

### Measures

We measured participant curiosity using the Five-Dimensional Curiosity Scale (24). This scale consists of 25 survey questions that evaluate five curiosity dimensions: deprivation sensitivity, joyous exploration, stress tolerance, social curiosity, and thrill-seeking. For each dimension, participants are asked to rate five statements on a 1 (“Does not describe me at all”) to 7 (“Completely describes me”) scale. These subscales’ reliability (Cronbach’s *α*) was good (α>0.75) for all five dimensions (see SI Appendix). Thus, we averaged the responses for each curiosity dimension and used them as our curiosity measures for the analysis.

### Experimental procedure

Once the study began, participants sat at a computer and gave informed consent using a digital form. Next, after a brief training session, participants made a total of 15 selections for their preferred books by clicking one cell on the 15 × 15 decision grid. After each selection, the corresponding book synopsis (∼150 words; matched to the average length of GoodReads metadata to simulate the information environment of real-world book discovery) was displayed, and participants were asked to evaluate how much they enjoyed the story on a 9-point Likert scale ranging from 1 (extremely dislike) to 9 (extremely like). We note that in this experimental task, participants were instructed to choose and read books, but they were effectively selecting and reading book synopses, not the full text, as in the real-world setting. While this synopsis-based selection task implies a lower opportunity cost than real-world purchasing, this design was chosen to balance experimental feasibility with ecological realism: it prevents participant fatigue while preserving the core cognitive structure of the sequential selection process to navigate a semantic space to maximize subjective value based on limited information cues. After the book selection task, participants were redirected to the Qualtrics platform to answer questions to measure their trait curiosity (50) and demographics, including age, gender, and race.

### Computational model fitting and evaluation

For the real-world dataset, we compared the predictive performance of two models *GP* and *UCB* with a default set of parameters (λ=1,τ=1,β=1), following a prior study (3). This model comparison method allows us to identify how well the generalization and exploration computational mechanisms capture real-world book selection patterns over nearly two million records.

For the experimental data, following (4), we used the cross-validated maximum likelihood estimation method to estimate a set of parameters ($\lambda ,\tau ,\beta_{\mathrm{bonus}}$) for each subject independently, which allows us to conduct further individual-level analysis. We used a Scipy (51) implementation of the global optimization differential evolution method, which effectively balances computational efficiency with optimization accuracy while accommodating parameter bounds, to optimize the likelihood objective function, defined as the sum of the log likelihood for all leave-one-out predictions. Since the differential evolution method is nondeterministic (each repetition might generate slightly different parameter estimation), we repeated the parameter estimation 100 times for each subject and took the average as the parameter estimates.

Finally, model performance was evaluated based on the predictive accuracy of each model’s leave-one-out predictions. We computed a pseudo-$R^{2}$ measure, which normalizes the log loss prediction error (*logL*) of model *M* with that of a random model $M_{\mathrm{rand}}$, which assumes a uniform distribution of option selection shown in Eq. 1.


(1) $$R^{2}=1-\frac{\log\;L(M)}{\log\;L(M_{\mathrm{rand}})}.$$

Here, $R^{2}>0$ indicates a prediction accuracy better than the null model, since $\log\;L(M)<\log\;L(M_{\mathrm{rand}})$, while $R^{2}\leq 0$ indicates a poor predictive accuracy worse than or equal to chance prediction.

## Supplementary Material

pgag226_Supplementary_Data

## Data Availability

The data supporting this study’s findings are publicly available. The Amazon book rating data are from the Amazon Review Data (https://cseweb.ucsd.edu/∼jmcauley/datasets/amazon_v2/)(17). The experimental behavioral data are available on OSF (https://osf.io/uak97/?view_only=80b0fdb3859545dd9760d4f60f257878). The code necessary to reproduce the experimental paradigm and the analysis code to reproduce the analyses are publicly available on GitHub (https://github.com/cogcommscience-lab/sequential_book_selection).

## Preprints

A preprint of this article is published at https://www.researchsquare.com/article/rs-5442881/v1.
