# Supplementary material for: Individual curiosity modulates exploration in sequential book selection
Source: PNAS Nexus. 2026 Jun 22;5(7):pgag226. doi: 10.1093/pnasnexus/pgag226 (PMC13326943; doi:10.1093/pnasnexus/pgag226)
Supplement: pgag226_Supplementary_Data [file pgag226_supplementary_data.pdf]

Supporting Information for

Individual Curiosity Modulates Exploration in Sequential Book Selection

Xuanjun Gong, Erie Boorman, Cuihua Shen and Richard Huskey

Xuanjun Gong.
E-mail: xjgong@tamu.edu

This PDF file includes:

Supporting text
Figs. S1 to S5
Tables S1 to S13
SI References

Supporting Information Text

Descriptive characteristics of book selection dataset

Experimental Stimulus Selection. The experimental decision task presents a multi-armed bandit task with 225 book options arranged in a 15x15 grid. We selected these 225 books in the following way: (A) we created a subsample of books with an average rating higher than 4 and more than 20 ratings in the GoodReads metadata, (B) we further subsampled books to include books that have a synopsis longer than 600 characters and shorter than 1200 characters, (C) we randomly sampled 25 books for each genre in a genre list consisting 18 common book genres, (D) the final selections were made based on synopsis quality.

Experimental Stimulus Selection and Validation. We collected perceived pairwise similarities among 22 sampled books from 248 participants recruited from Prolific. We randomly drew 15 pairs of book synopses for each participant and asked participants to read the synopses and evaluate the similarities between the book pairs. For each pair of these 22 books, we averaged participants' perceived similarity responses (ranging from 1 to 9) and constructed a 22x22 distance matrix. In addition, we also constructed another two 22x22 distance matrices with distance metrics calculated as the Euclidean distance between semantic embedding vectors of the 22 books and the distance metrics calculated as the Euclidean distance between 22 books in the grid space. We compared the two distance matrices and evaluated the association between these two distance metrics using the Mantel test (1). We conducted the Mantel test using 10,000 permutations on Pearson correlation metrics and conducted two-tailed significance tests. The results are shown in Table S1 and Fig. S1.

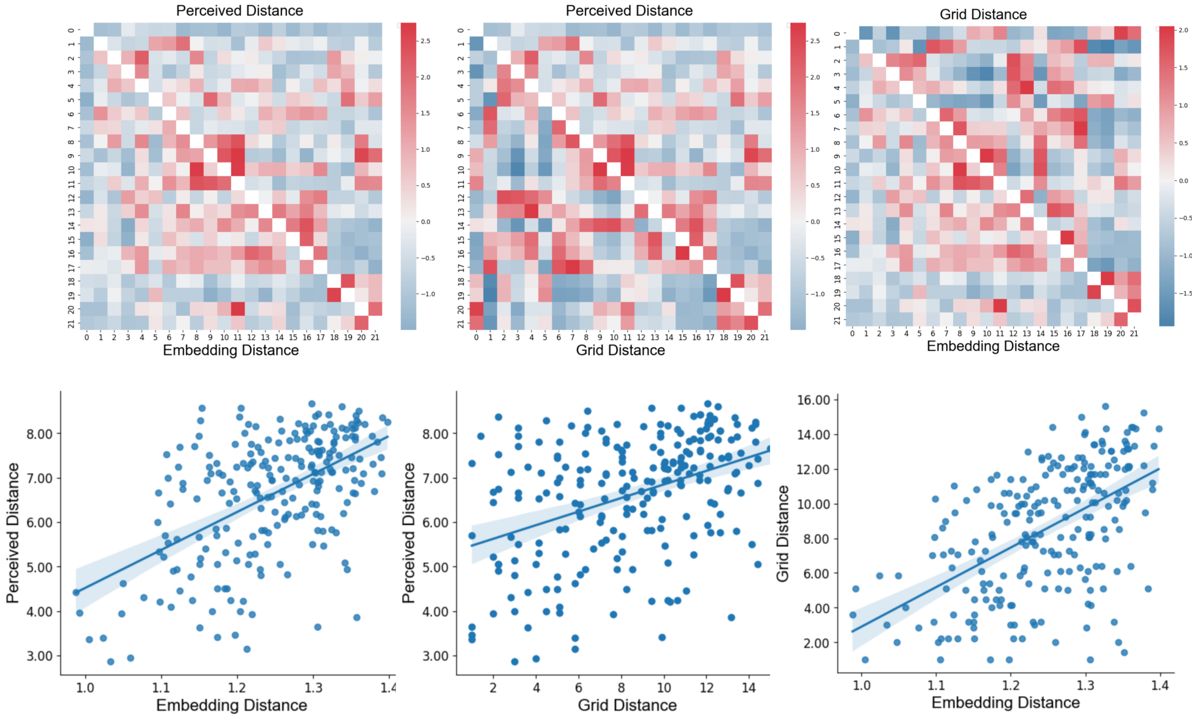

Fig. S1. Validation of semantic embeddings of books. Upper Panel: Using different distance metrics, the heatplots of the pairwise distance between sampled 22 books. The upper matrix and lower matrix encode different distance metrics. Color encodes normalized distance metrics. Bottom Panel: The regression plots of pairwise associations among three distance metrics. Columns represent different pairs of distance metrics: Semantic Embedding Euclidean Distance - Subject Perceived Distance (Left), Grid Space Distance - Subject Perceived Distance (Middle), and Semantic Embedding Euclidean Distance - Grid Space Distance (Right).

Table S1. Mantel test results to evaluate the association among different distance metrics (Euclidean).

Pair of distance metrics	r	Z	Empirical p-value
Perceived distance - Semantic Embedding distance (Euclidean)	0.546	7.247	<0.001
Perceived distance - Grid distance	0.410	6.199	<0.001
Semantic Embedding (Euclidean) - Grid distance	0.544	8.188	<0.001

Testing an alternative explanation: Learning to Choose Better Books or Enjoy Books More Over Time?. We found that people choose more favorable books over time, as shown by the increasing trend of book ratings as the number of books read increases. However, alternative explanations exist such that people are not learning to choose better books but are just becoming more favorable toward books as a function of time. This phenomenon, known as coherency maximization (2), is common in purchase behavior such that the more people purchase a product, the more they will like it. To test this alternative explanation, we combined two independent datasets (people's selection histories and GoodReads rating data) and found that there is a positive association between the average rating of books (scrapped from GoodReads) and people's number of past reads ($\beta = 0.114, SE = 0.002, Z = 60.485, p < 0.001, 99\%CI = [0.110, 0.117]$), shown in Fig. S2. Thus, we show that people are not just increasingly liking the selected book but are learning to select better books.

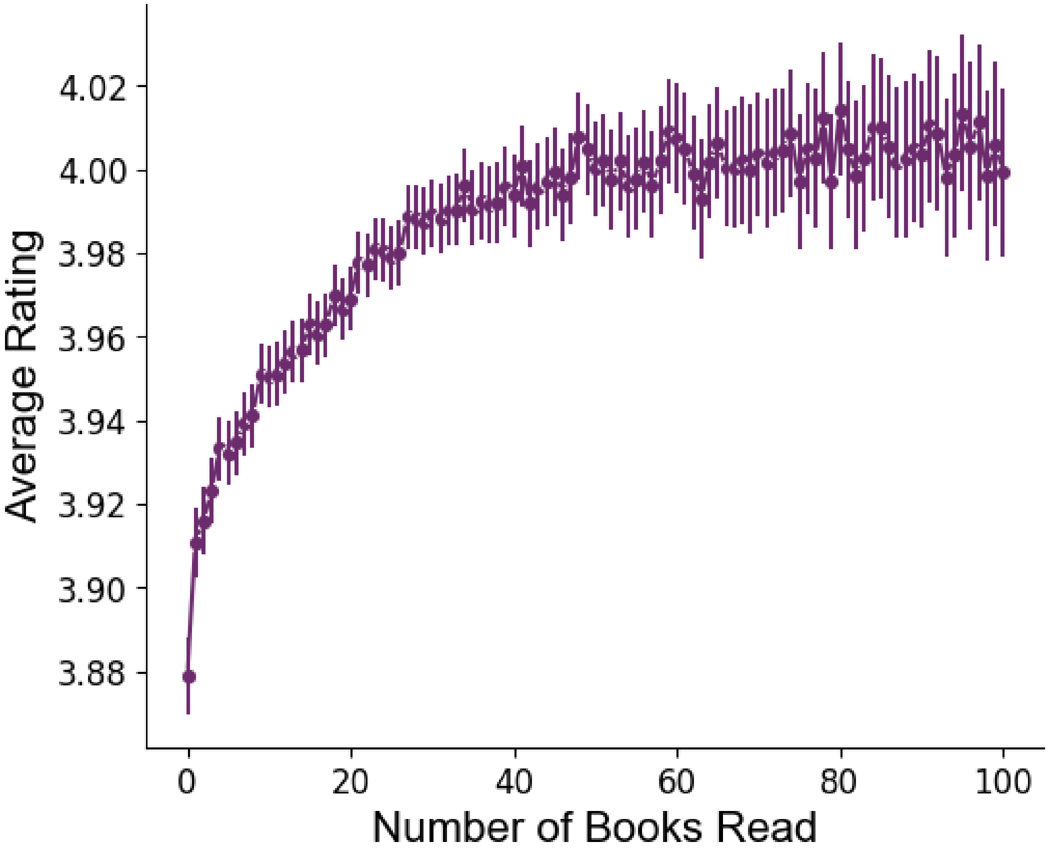

Fig. S2. The lineplot of book average rating (scrapped from GoodReads metadata) by the number of books read in the past.

Sensitivity Analysis: Mixed Effect Model for Exploration Distance while controlling for average rating

In our main analysis, we found a positive relationship between the rating variance and people's explore distance. However, we also identified a moderate negative correlation between the rating variance of books and their average rating. Therefore, it is possible that the positive relationship between rating variance and people's exploration distance is confounded by the average rating of the next choice. To address this issue, we estimated a mixed-effects regression model with both random slopes and random intercepts to check the robustness of our findings while controlling for the average rating of the books. The results, as shown in Table S2, indicate that the positive relationship between the rating variance and explore distance is robust to the average rating.

Table S2. Result of mixed effect model regression on explore distance controlling for average rating.

	Standardized Coefficient	SE	Z	$P > (\|Z\|)$
Current rating variance	0.020	0.002	9.777	< 0.001
Current average rating	-0.102	0.002	-59.438	< 0.001

Sensitivity Analysis: Mixed Effect Model for Exploration Distance and Reading Enjoyment

We conducted a sensitivity analysis using a mixed-effect model for the exploration distance in the real-world dataset, with a random intercept and random slopes for each subject in the dataset. Our main analysis found that people's exploration distance is associated with (A) the number of past reads, (B) the preceding reading enjoyment, (C) the number of following book reviews, and (D) the variance of following book reviews. Thus, in the mixed effect regression model, we specified fixed effects for these four variables and their corresponding random effects to control for individual differences (Equation 1, where j denotes the participant and i denotes the specific book choice within the sequence). Here, we report the model fitting standardized regression coefficient estimates for the fixed effects in Table S3.

$$\begin{aligned} ExploreDistance_{i,j} \sim \beta_0 + \beta_1 \#ofPastReading_{i,j} + \beta_2 Enjoyment_{i,j} + \\ \beta_4 VarianceRating_{i,j} + \beta_5 log(RatingSize_{i,j}) + \\ \gamma_{0,j} + \gamma_{1,j} \#ofPastReading_{i,j} + \gamma_{2,j} Enjoyment_{i,j} + \\ \gamma_{3,j} * VarianceRating_{i,j} + \gamma_{4,j} * log(RatingSize_{i,j}) + \\ \epsilon_{i,j} \end{aligned} \quad [1]$$

Table S3. Result of mixed effect model regression on explore distance in the real-world dataset.

	Standardized Coefficient	SE	Z	$P > (\|Z\|)$
Sequential order	-0.022	0.002	-9.598	< 0.001
Preceding reading enjoyment	-0.068	0.001	-61.101	< 0.001
Current rating variance	0.016	0.002	8.031	< 0.001
Current log number of ratings	-0.068	0.002	-37.665	< 0.001

Similarly, we fit the mixed-effect model for exploration distance in the experimental dataset. Note that the number and the variance of book reviews were not shown to the participants, we dropped these two variables in the model for experimental data, while adding people's curiosity measures to the model. Thus, we specified the mixed effect model with a random intercept and random slopes for each subject in the dataset and fixed effect variables as the sequential order of the exploration, preceding reading enjoyment, and the five dimensions of trait curiosity as independent variables (Equation 2). We report the model fitting standardized regression coefficient estimates in Table S4.

$$\begin{aligned} ExploreDistance_{i,j} \sim \beta_0 + \beta_1 \#ofPastReading_{i,j} + \beta_2 Enjoyment_{i,j} + \\ \beta_3 CuriosityJE_j + \beta_4 CuriosityTS_j + \beta_5 CuriosityDS_j + \\ \beta_6 CuriosityST_j + \beta_7 CuriositySC_j + \gamma_{0,j} + \\ \gamma_{1,j} \#ofPastReading_{i,j} + \gamma_{2,j} Enjoyment_{i,j} + \\ \epsilon_{i,j} \end{aligned} \quad [2]$$

Table S4. Result of mixed effect model regression on explore distance in the experimental dataset.

	Standardized Coefficient	SE	Z	$P > (\|Z\|)$
Sequential order	-0.154	0.022	-6.997	< 0.001
Preceding reading enjoyment	-0.315	0.018	-17.383	< 0.001
Joyous Exploration	0.082	0.035	2.319	0.020
Deprivation sensitivity	-0.041	0.033	-1.260	0.208
Stress tolerance	-0.039	0.034	-1.120	0.263
Thrill Seeking	0.021	0.036	0.585	0.559
Social Curiosity	-0.040	0.031	-1.304	0.192

Furthermore, our main analysis found people's explore distance is associated with their following reading enjoyment, and there exists an interaction effect between joyous exploration and explore distance on people's following reading enjoyment. Thus, in this sensitivity analysis, we fitted a mixed-effect regression model on people's reading enjoyment with independent variables including (1) preceding explore distance, (2) joyous exploration, and (3) an interaction effect between joyous exploration and preceding explore distance. Similar to the mixed effect model for the explore distance mentioned above, we specified an additional fixed effect variable as the sequential order of the exploration to account for the potential trend effect (Equation 3). We report the results in Tables S5.

$$Enjoyment_{i,j} \sim \beta_0 + \beta_1 \#ofPastReading_{i,j} + \beta_2 PreviousExploreDistance_{i,j} +$$
$$+\beta_3 CuriosityJE_j + \beta_4 PreviousExploreDistance_{i,j} * CuriosityJE_j +$$
$$\gamma_{0,j} + \gamma_{1,j} \#ofPastReading_{i,j} +$$
$$\epsilon_{t,j} \quad [3]$$

Table S5. Result of random intercept mixed effect model regression on reading enjoyment in the experimental dataset.

	Standardized Coefficient	SE	Z	$P > (\|Z\|)$
Sequential order	0.083	0.018	4.625	< 0.001
Preceding explore distance	-0.058	0.017	-3.534	< 0.001
Joyous Exploration	0.146	0.034	4.347	< 0.001
Preceding explore distance * Joyous Exploration	0.033	0.017	1.990	0.047

Sensitivity Analysis: Analysis on readers with fewer reading and rating records

Our main analysis focuses on readers with more than 30 reading and rating records, potentially prioritizing a sub-population of highly engaged Amazon readers compared to the general reader population. Therefore, we conducted a sensitivity analysis focusing on readers with less than 30 but more than 10 records (in order to maintain a sufficient amount of records to analyze the sequential reading patterns). In this subset data, on average, each reader left 14.3 ($SD = 4.8$) records. Consistent with our main results, readers' ratings increase as they read more books ($\beta = 0.032, SE = 0.001, Z = 50.103, p < 0.001, 95\%CI = [0.032, 0.033]$). The results of the mixed-effect model for exploration distance, following Equation 1, are reported in Table S6 and are consistent with our main results. Finally, the computational model results are also consistent with our main results, with the GP model yields a model accuracy higher than the random model ($R^2 = 0.007, Z = 50.040, p < 0.001, 99\%CI = [0.007, 0.007]$), and UCB model yields a model accuracy higher than the GP model ($R^2 = 0.042, Z = 1111.132, p < 0.001, 99\%CI = [0.041, 0.042]$).

Table S6. Sensitivity analysis result of mixed effect model regression on explore distance in the real-world dataset focusing on readers with fewer than 30 records.

	Standardized Coefficient	SE	Z	$P > (\|Z\|)$
Sequential order	-0.021	0.001	-28.630	< 0.001
Preceding reading enjoyment	-0.043	0.001	-68.029	< 0.001
Current rating variance	0.014	0.001	21.968	< 0.001
Current log number of ratings	-0.078	0.001	-110.536	< 0.001

Sensitivity Analysis: Analysis on verified reading and rating records

We acknowledge that the real-world Amazon dataset includes records not associated with a verified purchase. These unverified entries may represent less reliable signals or reflect reading experiences with lower financial commitment. To address this, we conducted a sensitivity analysis exclusively on verified purchase records. Consistent with our main results, reading enjoyment ratings increased as a function of reading history ($\beta = 0.027, SE = 0.002, Z = 12.519, p < 0.001, 95\%CI = [0.023, 0.032]$). The mixed-effect model for exploration distance (Equation 1) also yielded results largely consistent with the primary analysis (see Table S7). Finally, computational modeling confirmed the robustness of our findings: the GP model outperformed the random baseline ($R^2 = 0.013, Z = 22.281, p < 0.001, 99\%CI = [0.012, 0.015]$), and the UCB model significantly outperformed the GP model ($R^2 = 0.109, Z = 413.501, p < 0.001, 99\%CI = [0.107, 0.110]$).

Table S7. Result of mixed effect model regression on explore distance in the real-world dataset for verified records.

	Standardized Coefficient	SE	Z	$P > (\|Z\|)$
Sequential order	0.010	0.002	6.648	< 0.001
Preceding reading enjoyment	-0.031	0.001	-35.293	< 0.001
Current rating variance	0.010	0.001	13.017	< 0.001
Current log number of ratings	-0.073	0.001	-71.640	< 0.001

Sensitivity Analysis: Analysis with cosine distance metrics

Our main analysis was conducted using Euclidean distance metrics computed from semantic embeddings of the book synopses. To illustrate the robustness of this distance metric method, we conducted a sensitivity analysis using the cosine distance metric (3). The results are robust regardless of distance metric choice. The results remained consistent: reading enjoyment

ratings increased as a function of reading history ($\beta = 0.052, SE = 0.002, Z = 25.357, p < 0.001, 95\% CI = [0.048, 0.056]$). The mixed-effects model for exploration distance (Equation 1) also yielded results aligning with the main analysis (Table S8). Furthermore, computational model comparisons confirmed the robustness of these mechanisms: the GP model outperformed the random baseline ($R^2 = 0.014, Z = 30.439, p < 0.001, 99\% CI = [0.013, 0.016]$), and the UCB model significantly outperformed the GP model ($R^2 = 0.106, Z = 525.057, p < 0.001, 99\% CI = [0.104, 0.107]$).

We further validated the cosine distance metric using a Mantel test (1) to assess its alignment with human-rated similarity. Significance was determined via 10,000 permutations of the Pearson correlation coefficient. The results were effectively identical to those obtained using Euclidean distance ($r = 0.541, Z = 7.195, p < 0.001$).

Table S8. Result of mixed effect model regression on explore distance measured by cosine distance metric in the real-world dataset.

	Standardized Coefficient	SE	Z	$P > (\|Z\|)$
Sequential order	-0.014	0.003	-5.645	< 0.001
Preceding reading enjoyment	-0.063	0.004	-16.316	< 0.001
Current rating variance	0.017	0.005	3.786	< 0.001
Current log number of ratings	-0.053	0.004	-12.563	< 0.001

Computational modeling

Following previous studies(4, 5), we constructed two computational models: a Gaussian Process (GP) regression model for the reward generalization mechanism and an Upper Confidence Bound (UCB) model for the directed exploration mechanism.

Gaussian process regression. In principle, GP(6) formalizes a value function f that takes the options' semantic features x in a multi-dimensional space (semantic embeddings in real-world data and grid embedding in experiment data) as inputs and outputs a scalar value as the expected reward. This function is modeled as a multivariate Gaussian.

$$f \sim GP(m(x), k(x, x')) \quad [4]$$

$$m(x) = E[f(x)] \quad [5]$$

$$k(x, x') = E[(f(x) - m(x))(f(x') - m(x'))] \quad [6]$$

Here $m(x)$ denotes the expected value for the value function of option x, and $k(x, x')$ encodes the value covariance between two options x and x'.

Consider a sequence of observations of book selections $\boldsymbol{X} = [x_1, x_2, ..., x_t]$ and their corresponding rewards $\boldsymbol{y} = [y_1, y_2, ..., y_t]$ from time 1 to time t. GP regression uses the Bayesian principle to generate the posterior predictions of a target option x^*, by computing its posterior mean $m(x^*)$ and variance $v(x^*)$ conditional on observations $\boldsymbol{X}, \boldsymbol{y}$:

$$m(x^*|\boldsymbol{X}, \boldsymbol{y}) = \boldsymbol{k_*}^T(\boldsymbol{K} + \sigma^2\boldsymbol{I})^{-1}\boldsymbol{y} \quad [7]$$

$$v(x^*|\boldsymbol{X}, \boldsymbol{y}) = \boldsymbol{k_*}^T(\boldsymbol{K} + \sigma^2\boldsymbol{I})^{-1}\boldsymbol{k_*}^T \quad [8]$$

Here $\boldsymbol{k_*}$ denotes the $1 * t$ covariance matrix between input observations $\boldsymbol{X}$ and the target option x^*, $\boldsymbol{K}$ denotes the $t * t$ covariance matrix of the input observations $\boldsymbol{X}$, and σ^2 denotes the assumed noise from the observations. Here, we specify $\sigma^2 = 1$.

The covariance between two options is defined by a Radial Kernel function so that the posterior estimation depends on the Euclidean distance between target options and the input observations:

$$k(x, x') = exp(-\frac{||x - x'||^2}{2\lambda^2}) \quad [9]$$

In this way, the GP regression and the kernel function together formalize the reward generalization mechanism by assuming that similar options will generate similar reward outcomes. The length-scale parameter λ in the kernel function regulates this generalization process by controlling the smoothness of the generalization function, in a way such that $\lambda \to 0$ leads to zero generalization and independent value estimation among options, whereas $\lambda \to \infty$ leads to maximum generalization, such that the dependency of value estimation is linear to feature distances.

Value Function and Decision Function. The GP regression model simply considers that the value for a target option $Q_{GP}(x^*)$ equals its expected reward $m(x^*)$ as:

$$Q_{GP}(x^*) = m(x^*) \quad [10]$$

Conversely, the UCB model specifies the value for a target option $Q_{UCB}(x^*)$ as a weighted sum of the expected reward $m(x^*)$ and the square root of the variance of the reward estimation $v(x^*)$:

$$Q_{UCB}(x^*) = m(x^*) + \beta_{bonus}\sqrt{v(x^*)} \quad [11]$$

Here, the exploration bonus parameter β_{bonus} controls the extent of directed exploration, with higher β_{bonus} leading to a stronger bias towards options with high uncertainty.

Next, we specify the decision function for both models as a softmax function, which maps the estimated value for N available options to the probabilities of choosing an option x_i as $p(x_i)$:

$$p(x_i) = \frac{exp(Q(x_i)/\tau)}{\sum_j^N exp(Q(x_j)/\tau)} \quad [12]$$

Here, the random temperature parameter τ in this softmax controls the randomness in the probabilistic mechanism. $\tau \to 0$ leads to zero randomness, such that the highest-valued option is always chosen, whereas $\tau \to \infty$ leads to maximum randomness with a uniform probability of selecting any option.

A Challenge for Comparing Computational Models with Real-world Data. We recognize model performance comparisons depend on the relative exploration or exploitation tendencies between observed choices and alternatives. Consider, an empirical choice was observed and used to estimate the performance of models with or without directed exploration. If all the alternative options were high in estimated uncertainty, the observed empirical choice would become relatively more exploitative, thus favoring a model without directed exploration. Similarly, an exploitive option space for the available alternatives will bias the model comparison, favoring the model with a directed exploration mechanism.

As a result, information on the option space is vital for an unbiased comparison between exploration and exploitation models. However, we do not know such information for real-world data. Consistent with prior research (7), we specified an artificial alternative option with averaged features of all available options, but this fixed, arbitrary, and small option space inevitably introduces opaque biases in our comparison results for real-world data. We note that our results from experiment data are not subject to this issue because the alternative option space is always known. Thus, our parallel analysis of real-world and experimental data helps us overcome this challenge.

Multiple Regression on Exploration Parameters

We applied multiple regression models on the exploration parameters (i.e., τ, β_{bonus}) with curiosity dimensions as independent variables while controlling for participants' age, as specified in Equation 13. The result of the regression model is reported in Supplementary Table S9.

$$\begin{aligned}\tau, \beta_{bonus} \sim \beta_0 + \beta_1 Age + \beta_2 CuriosityJE + \beta_3 CuriosityTS + \\ \beta_4 CuriosityDS + \beta_5 CuriosityST + \beta_6 CuriositySCj + \epsilon\end{aligned} \quad [13]$$

Table S9. Result of multiple regression on decision parameters.

	Bonus β_{bonus}				Temperature τ			
	Standardized Coefficient	SE	$t(238)$	$P > (\lvert t \rvert)$	Standardized Coefficient	SE	$t(238)$	$P > (\lvert t \rvert)$
Intercept	-0.000	0.063	-0.000	1.000	0.000	0.063	0.000	1.000
Age	-0.030	0.068	-0.442	0.659	0.0241	0.067	0.359	0.720
**Joyous-Exploration**	**0.229**	**0.081**	**2.824**	**0.005**	**-0.160**	**0.080**	**-1.983**	**0.049**
**Thrill-Seeking**	-0.064	0.083	-0.771	0.441	**0.200**	**0.082**	**2.440**	**0.015**
Deprivation-Sensitivity	-0.061	0.075	-0.816	0.416	-0.104	0.074	-1.399	0.163
Stress-Tolerance	0.019	0.082	0.228	0.820	-0.024	0.082	-0.296	0.768
Social-Curiosity	0.010	0.071	0.137	0.891	0.064	0.070	0.915	0.361

In addition, we plotted the bi-variate pairwise correlation matrix among the dependent variables and independent variables in our specified regression model in Fig. S3.

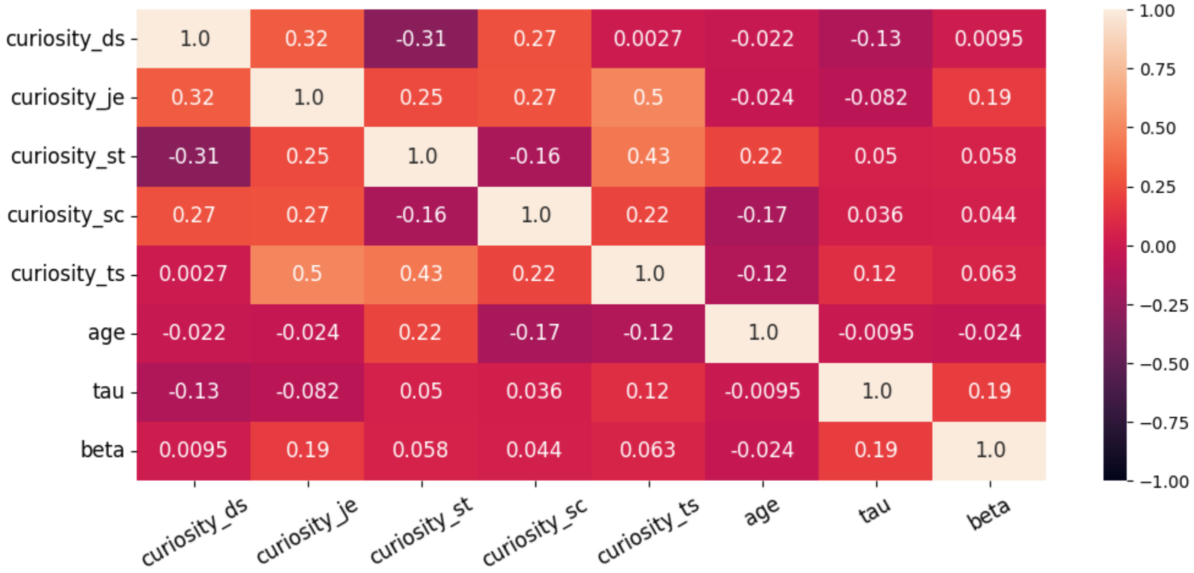

Fig. S3. The bivariate Pearson correlation matrix of individual-level variables.

Sensitivity Analysis on Multiple Regression. We conducted a sensitivity analysis for the multiple regression on the exploration parameters after the removal of the non-significant variables. The results are reported in Table S10 for exploration bonus β_{bonus} and Table S11 for random temperature τ.

Table S10. Result of regression model on log exploration bonus β_{bonus} after removal of non-significant variables.

	Standardized Coefficient	SE	$t(243)$	$P > (\|t\|)$
Intercept	-0.000	0.063	-0.000	1.000
Joyous Exploration	0.186	0.063	2.945	0.004

Table S11. Result of multiple regression on log random temperature τ after removal of non-significant variables.

	Standardized Coefficient	SE	$t(242)$	$P > (\|t\|)$
Intercept	0.000	0.063	0.000	1.000
Joyous Exploration	-0.1893	0.072	-2.613	0.010
Thrill Seeking	0.2155	0.072	2.974	0.003

Sensitivity Analysis with Censored Regression. We recognized that a considerable portion of the parameter estimation for the exploration bonus β_{bonus} reaches the boundary close to 0. This issue might hamper the regression coefficient estimation due to a non-normal error distribution. Thus, we fit a censored regression on exploration bonus β_{bonus}, which assumes the dependent variable is censored at boundaries near 0. The result remains consistent with the multiple regression results and reported in Table S12.

Table S12. Result of censored regression on log exploration bonus β_{bonus}.

	Standardized Coefficient	SE	$t(237)$	$P > (\|t\|)$
Intercept	-0.051	0.070	-0.737	0.461
Age	-0.029	0.074	-0.394	0.693
**Joyous Exploration**	**0.262**	**0.089**	**2.939**	**0.003**
Thrill Seeking	-0.068	0.091	-0.755	0.450
Deprivation Sensitivity	-0.068	0.082	-0.823	0.410
Stress Tolerance	0.018	0.091	0.193	0.847
Social Curiosity	0.024	0.077	0.313	0.754

Parameter Estimation Stability. We estimated the parameters using a global optimization method—the differential evolution algorithm. This global optimization method is non-deterministic, so we repeated the parameter estimation 100 times and took the average as our final parameter estimate. Fig. S4 plots the within-subject variance of these 100 repetitions of parameter estimations and shows that there is very small within-subject variability for both parameter estimates, thus indicating that our parameter estimation method is stable.

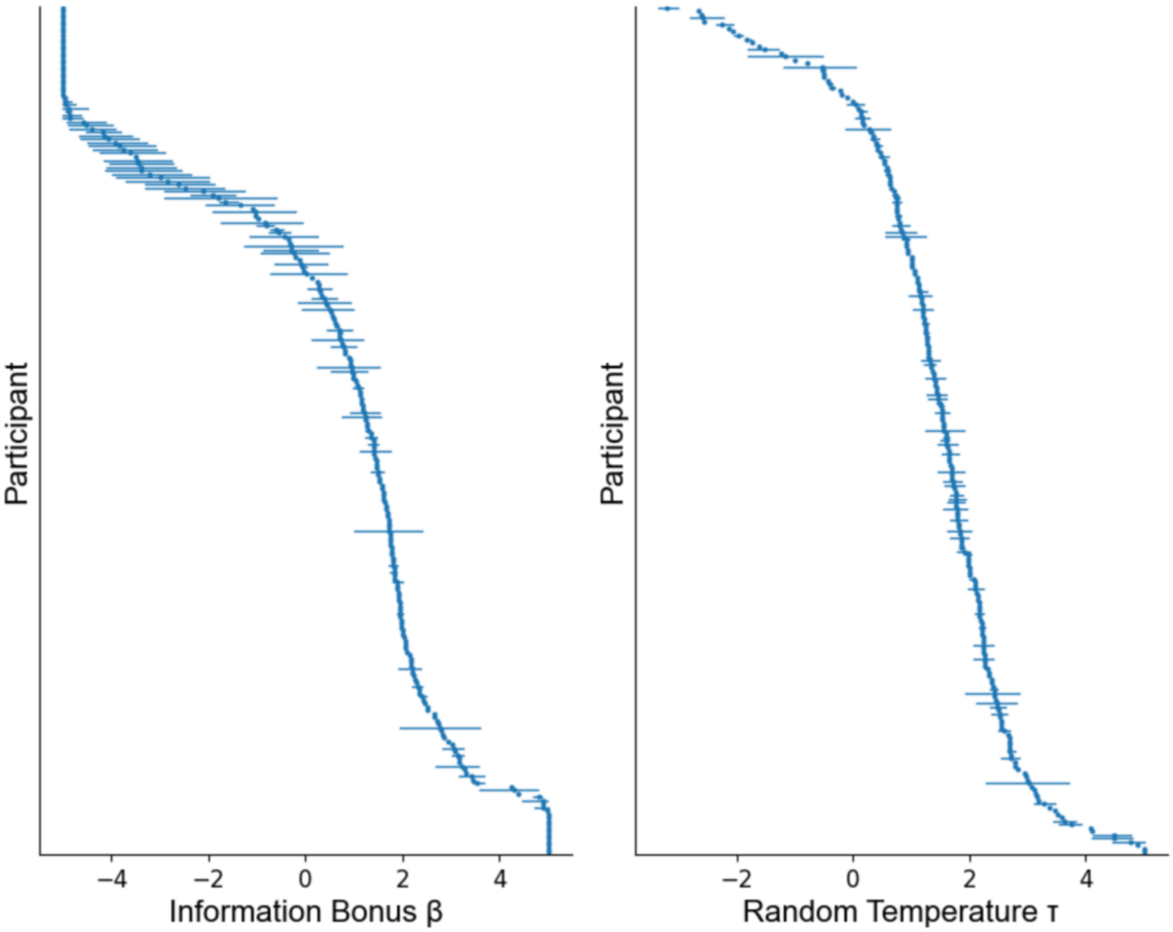

Fig. S4. Parameter estimations across different optimization repetition and different participants. The y-axis encodes the participants ordered by a mean estimate of the parameter in descending order, while the x-axis encodes the exploration parameter for exploration bonus β_{bonus} (left) and random temperature τ (right). The horizontal line encodes the 99% confidence interval of 100 repetitions of parameter estimations.

Cronbach's α for Curiosity Dimensions. To estimate the validity of the five-dimension curiosity scale, we measured Cronbach's α statistic for each of the five dimensions including deprivation sensitivity, joyous exploration, stress tolerance, social curiosity, and thrill-seeking. The results are shown in Table S13 below. All dimensions of curiosity have a Cronbach's α larger than 0.75, indicating good reliability of the scale.

Table S13. Cronbach's α for the five curiosity dimensions

	Cronbach's α	95% Confidence Interval
Deprivation Sensitivity	0.822	[0.788, 0.853]
Joyous Exploration	0.788	[0.742, 0.827]
Stress Tolerance	0.854	[0.823, 0.881]
Social Curiosity	0.833	[0.797, 0.864]
Thrill Seeking	0.791	[0.747, 0.830]

Instructions in Experimental Paradigm.

Experimental Instructions and Script

In this experiment, the task includes three parts. In the first part, you will be shown different books, and asked to choose which you would like to read. Second, after you have made a choice on a book, you will be shown a summary that introduces the plot and story of the book that you have chosen. Third, after you have read the book summary, you need to respond how much you enjoyed reading the book summary. Then, you need to indicate how certain you are for your response on your enjoyment.

These three parts of the task will repeat 15 times. There will be a training session that helps you learn how to do this task. In the first part, we will show you a set of book options that are displayed in a grid. The grey boxes in the grid will represent different book options. For example, you may see a grid of book options like the 3x3 grid on the right. In the actual task, you will see a much larger grid. Importantly, the distance between boxes in the grid represents similarities between books. The closer two boxes are, the more similar the two books are. To choose a book that you would like to read, you need to click a box to select the corresponding book. For example, to choose the book on the left bottom of the grid, you need to click the box that is displayed at the left bottom of the grid. We have marked the box with a red outline. Now click the box to select this book. After you have selected the book option, you will read the summary of the book that introduces the plot and story of the book.

For example, you need to read the summary of the book that you have just selected. After you have read the book summary, you need to respond to the following question: How much did you like the book summary you just read? Please click the button to choose your response. The red buttons on the right are for books that you really like. The blue buttons on the left are for books that you really dislike. You can use all buttons inbetween.

> extremely disliked - very disliked - somewhat disliked - slightly disliked - neither liked nor disliked - slightly liked - somewhat liked - very liked - extremely liked

Then, you need to respond to the following question: Please indicate how certain you are about your choice. As a reminder, you just indicated that you `[PLACEHOLDER]` the book. Please click the button to choose your response.

> extremely uncertain - very uncertain - somewhat uncertain - slightly uncertain - neither certain nor uncertain - slightly certain - somewhat certain - very certain - extremely certain

Now you have finished one trial of the task. There are a total of 15 trials in the actual task. Your task is to choose books that maximize your reading enjoyment. After you have indicated your enjoyment on the selected book option, we will mark the book option with colors based on your preference. Reddish color indicates liked, Bluish color indicates disliked. Remember, the closer two books are, the more similar they are. For example, the book that has a green box outline is more similar to the book that has a red box outline, compared to the book with a blue box outline. This is because the green box is closer to the red box, compared to the blue box.

Now, you have completed the training session! When you are ready, you may press the spacebar on your keyboard to start the experiment!

Experiment (see Figure S5) Click a grey box to select a book to view. Make a selection that maximizes your reading enjoyment. This is the book summary.

`[BOOK SYNOPSIS]`

If you have finished reading this book summary, press the spacebar on your keyboard to continue.

How much did you like the book summary you just read? Please click the button to choose your response.

> extremely disliked - very disliked - somewhat disliked - slightly disliked - neither liked nor disliked - slightly liked - somewhat liked - very liked - extremely liked

Please indicate how certain you are about your choice. As a reminder, you just indicated that you `[PLACEHOLDER]` the book. Please click the button to choose your response.

> extremely uncertain - very uncertain - somewhat uncertain - slightly uncertain - neither certain nor uncertain - slightly certain - somewhat certain - very certain - extremely certain

---

Attention Check In the following task, you will complete a recall task. You will indicate whether or not you have read the summary of a book in the previous task. If you have read the summary, please click the button "Yes, I have read it". If not, please click the button "No, I have not read it". Please note that this is just

a recall task. We do NOT use this recall check to reject your submission. Press the spacebar on your keyboard to continue.

Have you read this book summary in the previous task?

[BOOK SYNOPSIS]

Dismissal
Thank you! You have completed this task. Now, wait for 5 seconds. You will be soon redirected to a survey web page.

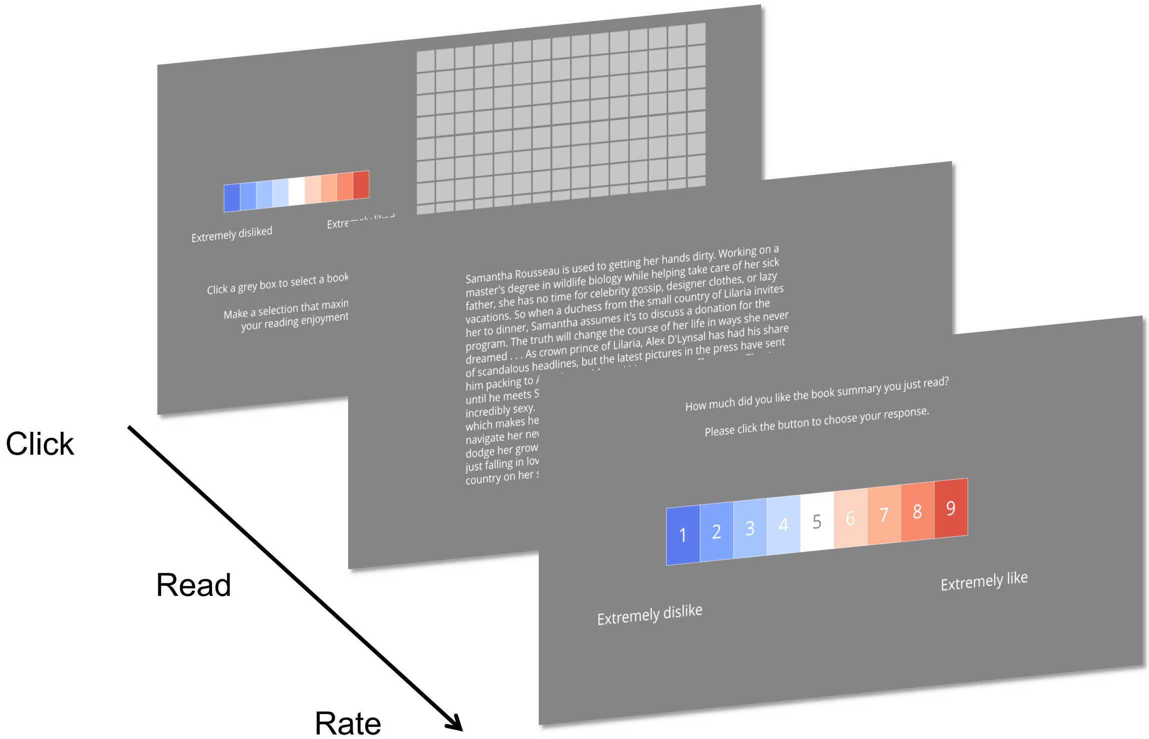

Fig. S5. Illustration of the experimental paradigm. Participants complete the sequential book selection task following a paradigm where they sequentially click an option on the option grid, read the book synopsis, and evaluate how they enjoyed the book, for a total of 15 selections.

The Five Dimensional Curiosity Scale Used in Experiment.

The 5DSC items (8)

Below are statements people often use to describe themselves. Please use the scale below to indicate the degree to which these statements accurately describe you. There are no right or wrong answers.

1 – Does not describe me at all
2 – Barely describes me
3 – Somewhat describes me
4 – Neutral
5 – Generally describes me
6 – Mostly describes me
7 – Completely describes me

Joyous exploration:
I view challenging situations as an opportunity to grow and learn.
I am always looking for experiences that challenge how I think about myself and the world.
I seek out situations where it is likely that I will have to think in depth about something.
I enjoy learning about subjects that are unfamiliar to me.
I find it fascinating to learn new information.

Deprivation sensitivity:
Thinking about solutions to difficult conceptual problems can keep me awake at night.
I can spend hours on a single problem because I just can't rest without knowing the answer.
I feel frustrated if I can't figure out the solution to a problem, so I work even harder to solve it.
I work relentlessly at problems that I feel must be solved.
It frustrates me not having all the information I need.

Stress tolerance: (entire subscale reverse-scored)
The smallest doubt can stop me from seeking out new experiences.
I cannot handle the stress that comes from entering uncertain situations.
I find it hard to explore new places when I lack confidence in my abilities.
I cannot function well if I am unsure whether a new experience is safe.
It is difficult to concentrate when there is a possibility that I will be taken by surprise.

Social curiosity:
I like to learn about the habits of others.
I like finding out why people behave the way they do.
When other people are having a conversation, I like to find out what it's about.
When around other people, I like listening to their conversations.
When people quarrel, I like to know what's going on.

Thrill seeking:
The anxiety of doing something new makes me feel excited and alive.
Risk-taking is exciting to me.
When I have free time, I want to do things that are a little scary.
Creating an adventure as I go is much more appealing than a planned adventure.
I prefer friends who are excitingly unpredictable.

References

1. N Mantel, The detection of disease clustering and a generalized regression approach. *Cancer Res.* **27**, 209–220 (1967).
2. PS Riefer, R Prior, N Blair, G Pavey, BC Love, Coherency-maximizing exploration in the supermarket. *Nat. Hum. Behav.* **1**, 1–4 (2017).
3. G Salton, A Wong, CS Yang, A vector space model for automatic indexing. *Commun. ACM* **18**, 613–620 (1975).
4. E Schulz, et al., Structured, uncertainty-driven exploration in real-world consumer choice. *Proc. Natl. Acad. Sci.* **116**, 13903–13908 (2019).
5. CM Wu, E Schulz, M Speekenbrink, JD Nelson, B Meder, Generalization guides human exploration in vast decision spaces. *Nat. Hum. Behav.* **2**, 915–924 (2018).
6. CE Rasmussen, CKI Williams, *Gaussian Processes for Machine Learning.* (The MIT Press), (2005).

7. E Schulz, et al., Structured, uncertainty-driven exploration in real-world consumer choice. *Proc. Natl. Acad. Sci.* **116**, 13903–13908 (2019).
8. TB Kashdan, et al., The five-dimensional curiosity scale: Capturing the bandwidth of curiosity and identifying four unique subgroups of curious people. *J. Res. Pers.* **73**, 130–149 (2018).
